# Hyperchaotic fractional-order image encryption with Knight’s tour scrambling for satellite imagery

**DOI:** 10.1038/s41598-026-58982-1

**Published:** 2026-06-29

**Authors:** Eyad Mamdouh, Amr Aboshousha, Wassim Alexan

**Affiliations:** 1 Communications Department, Faculty of Information Engineering and Technology, German University in Cairo, Cairo, 11835 Egypt; 2 Physics Department, Faculty of Basic Sciences, German University in Cairo, Cairo, 11835 Egypt

**Keywords:** Adaptive diffusion, Chaotic encryption, Fractional-order hyperchaos, Hyperchaotic Vaidyanathan system, Image cryptography, Knight’s tour, Remote sensing security, S-box design, Satellite imagery, Engineering, Mathematics and computing

## Abstract

Secure dissemination of high-resolution satellite imagery remains challenging because many image-tailored ciphers either (i) emphasize permutation-heavy designs without sufficiently strong, plaintext-adaptive nonlinearity, or (ii) provide strong security metrics but fall short on scalable, near-real-time performance and robustness assessment under realistic channel impairments. To address these gaps, this work proposes a three-stage chaos-chess hybrid encryption pipeline for color satellite images that couples fractional-order hyperchaotic key generation with lightweight algebraic mixing, dynamic substitution, and structured bit-level diffusion. First, multiple images are optionally augmented and each RGB channel is partitioned into $2\times 2$ pixel matrices that are mixed via invertible matrices derived from a 6D fractional-order hyperchaotic Vaidyanathan system, providing efficient confusion suitable for parallelization. Second, plaintext-sensitive S-boxes are constructed online from a 4D fractional-order hyperchaotic system and applied per channel to enhance nonlinearity and satisfy stringent criteria (NL $=108$, SAC $\approx 0.5$, low LAP and DAP). Third, the resulting bit-streams are diffused by traversing $8\times 8$ blocks using Knight’s Tour paths and XORing with 4D hyperchaotic key-streams to amplify avalanche propagation. Experiments on satellite and natural images demonstrate high ciphertext randomness (entropy $\approx 7.999$), strong differential resistance (NPCR $\approx 99.62\%$, UACI $\approx 30.95\%$), near-zero adjacent-pixel correlation (PCC $\approx 0$), and a large key space ($>2^{3827}$), while measured runtimes indicate suitability for real-time or near-real-time operation. Noise-like ciphertexts and lossless recovery are verified via visual, histogram, and DFT analyses, and robustness under occlusion and noise attacks (salt-and-pepper, Gaussian) is evidenced. The resulting modular design provides a scalable pathway for protecting remote sensing data and supports future integration with ROI-aware processing and hardware acceleration.

## Introduction

Satellite imagery plays a critical role in a wide array of domains, including environmental monitoring, urban planning, agriculture, military reconnaissance, and disaster management. The increasing availability and resolution of satellite data, powered by the proliferation of remote sensing technologies, have significantly enhanced decision-making capabilities across sectors^1^. However, as satellite images are transmitted, stored, and shared across various platforms, often over insecure or public channels, they become vulnerable to unauthorized access, tampering, and misuse^2^.

Ensuring the confidentiality, integrity, and authenticity of satellite images has thus become imperative, especially in sensitive applications involving national security or proprietary geospatial analysis^3^. Conventional cryptographic algorithms such as AES and RSA, while robust for general-purpose data protection, may not be efficient or adaptable for large-scale, real-time satellite image encryption due to their computational overhead and structural rigidity. Moreover, image data exhibits strong spatial redundancy and high correlation among neighboring pixels. These are characteristics that traditional encryption schemes are not optimized to handle^4^.

To address these limitations, researchers have explored various image-specific encryption approaches, particularly those capitalizing on the properties of chaos theory^5–9^. Chaotic systems are highly sensitive to initial conditions and exhibit pseudo-random behavior, making them well-suited for creating confusion and diffusion in image data. These properties enable the design of lightweight, fast, and secure encryption algorithms tailored to the unique statistical features of satellite imagery^10,11^.

Recent works have proposed a diverse set of techniques, including chaotic maps, DNA computing, transformation-based scrambling, and hybrid cryptographic models. Some approaches integrate object detection or region-of-interest (ROI) encoding to selectively encrypt sensitive image portions, thereby improving computational efficiency. Others combine chaos with biologically inspired methods like RNA or ECC-based key hiding to further enhance security. However, despite promising results, many of these methods lack standardization, real-time validation, or scalability to high-resolution datasets^12–24^.

The contributions of this work are:

- A multi-stage chaos-chess hybrid cipher tailored for satellite imagery, integrating fractional-order hyperchaotic keying, dynamic S-box substitution, and Knight’s Tour-guided diffusion.
- A plaintext-sensitive key schedule using coupled 6D, 4D fractional hyperchaotic systems, enhancing sensitivity and key diversity.
- A dynamic, plaintext-related S-box construction method meeting strong cryptographic metrics (e.g., nonlinearity, SAC, low LAP/DAP).
- A $2\times 2$ matrix mixing step that provides lightweight, algebraic confusion suited for high-resolution images and parallel hardware.
- A Knight’s Tour-based traversal strategy for block-wise diffusion on $8\times 8$ blocks, improving spread of local changes with low overhead.
- A comprehensive security evaluation showing near-ideal entropy, high NPCR and appropriate UACI, near-zero adjacent-pixel correlation, and a large key space.
- Robustness analyses under occlusion and common noise models (salt-and-pepper, Gaussian), demonstrating reliable decryption in degraded channels.
- Efficiency results indicating real-time or near-real-time performance, highlighting suitability for remote sensing pipelines.
- A modular design compatible with selective/ROI-aware encryption and scalable to multi-image streams.
- Implementation and analysis choices that facilitate future hardware acceleration and standards-aligned benchmarking.

This article is organized as follows. Section 2 reviews the related literature on satellite image encryption. Section 3 introduces the mathematical constructs, including the 4D and 6D fractional-order hyperchaotic systems and the Knight’s Tour. Section 4 presents the proposed algorithm (encryption and decryption). Section 5 reports the performance evaluation and robustness analyses. Finally, Sect. 6 concludes the paper and outlines future work.

## Related literature

This section first highlights recent advances in satellite image encryption, focusing on approaches that leverage chaotic systems, algebraic structures, hardware optimization, homomorphic encryption, and hybrid cryptographic techniques, each offering unique strengths in enhancing security, efficiency, and robustness. Next, it provides an appropriate justification for the utilization of fractional-order hyperchaos in this work.

### State-of-the-art

For starters, the authors of^12^ present a symmetric image encryption algorithm that uses dynamically generated S-boxes and P-boxes. The P-boxes are built using the Fisher-Yates shuffle guided by the JSMP chaotic map, while the S-boxes are created through modular transformation and dynamic permutation. The encryption operates over three color planes and applies substitution and permutation in multiple rounds, making the image highly resistant to structural analysis.

In^14^, the authors propose a hardware-based image encryption system for Earth observation satellites using a pipelined AES architecture combined with Hamming code. The AES algorithm is implemented with a T-Box structure to optimize hardware usage and speed, while Hamming coding is integrated after each AES round to detect and correct transmission errors. The system uses a reconfigurable key length of 128, 192, or 256 bits, and is deployed on a radiation-hardened FPGA, making it suitable for secure and reliable satellite image transmission.

While in^15^, the authors propose a two-phase image security system that combines watermark embedding and encryption for large satellite images. The watermarking phase applies a series of transformations, such as DWT, DORMT, Schur decomposition, and SVD, to embed a color watermark into a color host image. The second phase encrypts the watermarked image using a chaotic $e\pi$ map to ensure secure transfer and storage. An Aquila Optimization Algorithm is used to adaptively tune parameters for optimal watermark embedding, enhancing robustness and invisibility.

Unprecedentedly, in^16^, the authors propose an image encryption framework for satellite images by combining chaos theory with a chess-based pixel scrambling strategy. A 5D multi-wing hyperchaotic system is used to generate five random number streams. These streams control the random movements of two chess pieces—Castle and King—on a virtual board, where their positions determine pixel swap operations for confusion. After the scrambling phase, diffusion is applied by XORing the scrambled image with a random mask derived from the chaotic system. Plaintext sensitivity is ensured by modifying initial conditions using the SHA-256 hash of the input image.

The authors of^17^ propose a satellite image encryption scheme that combines chaotic systems with algebraic cryptographic techniques. The method begins with a permutation phase where pixel rows are shifted based on calculated values. Then, a diffusion phase uses XOR operations between alternating rows. A circulant matrix is used as a key for Hill cipher-based substitution. This is followed by further diffusion using secret keys generated from a hyperbolic tangent tent map. To enhance complexity, the Kronecker XOR product is applied for pixel expansion and additional scrambling. The result is a multi-layered encryption process with strong resistance against statistical and differential attacks.

In^18^, the authors propose a satellite image encryption algorithm that integrates a 7D complex chaotic system with RNA computing. The encryption process begins with generating multiple chaotic sequences using a 7D system, which are used for both scrambling and diffusion. The scrambling phase combines an Arnold transform with chaotic sequences to rearrange pixel positions. In the diffusion phase, pixel values are encoded into RNA sequences, then modified using RNA-specific operations, such as substitution, XOR, and complement, guided by chaotic sequences and a randomized RNA codon table. This dual-layered approach of spatial and value transformation enhances the algorithm’s complexity and resistance to attacks.

While in^19^, the authors propose an enhanced Blowfish encryption algorithm for electro-optical satellite images by replacing the traditional S-boxes with structures based on the Galois ring. The modified cipher reduces computational complexity by using three S-boxes instead of four, and processes 24-bit input blocks rather than the standard 32-bit blocks. This substitution increases nonlinearity and randomness, strengthening the confusion component while reducing encryption time. Experimental analysis shows that the proposed scheme achieves higher entropy, lower correlation, and better resistance against differential attacks compared to the standard Blowfish, achieving robust encryption in fewer rounds.

Moreover, in^20^, the authors propose an optical remote sensing image encryption algorithm (FMSMEA) specifically designed for sensitive maritime targets. The method employs YOLOv7 to detect sensitive objects such as warships, submarines, and aircraft carriers in sea-related scenes, enabling selective encryption. The encryption process uses a newly designed chaotic map, based on a discrete fractional-order memristor and sine map, to generate highly random sequences. The image is encrypted in two stages: first, by permuting the pixel positions using multi-level shuffling guided by chaotic sequences; second, by cross-channel diffusion, where each color channel influences the others. The algorithm exhibits strong security features, including high entropy, low correlation, robustness to noise and data loss, and resistance to differential and chosen-plaintext attacks.

While in^13^, the authors present a secure compression and encoding scheme for remote sensing images, integrating a novel Sin chaos paradigm, DNA transversion, and elliptic curve-based key hiding. The proposed method first generates plaintext-related keys using a scalable 2D-Sin2 chaotic system. Remote sensing images are then compressed using the discrete cosine transform and sparse representation. DNA encoding and dynamic transversion expand encoding diversity, while XOR operations with chaos-based sequences enhance diffusion. An elliptic curve “ring” strategy embeds encryption keys into the ciphertext image, ensuring secure key transmission. The scheme demonstrates robustness against statistical, differential, noise, and shearing attacks, and supports recovery even after $75\%$ data compression.

The authors of^21^ propose a Hybrid Block Scrambling and Somewhat Homomorphic Encryption (HBS2E) scheme for satellite image security. The encryption process combines Block Scrambling Encryption (BSE), which permutes and transforms pixel blocks using multiple secret keys, with Somewhat Homomorphic Encryption (SHE), allowing computations on encrypted data without decryption. This hybrid method protects images during transmission and enables secure classification. The encrypted images are decrypted only at the classification stage using the Modified Single Shot Multibox Detector (MSSMD) model, which is optimized by a Dynamic Hippopotamus (DH) algorithm. The proposed encryption system achieves high encryption ($98\%$) and decryption ($97\%$) accuracy with strong resistance against brute force and statistical attacks.

Moreover, in^22^, the authors propose a high-throughput multiple image encryption scheme aimed at real-time hardware use. It combines a 10D hyperchaotic system, an 8D hyperchaotic system, and a memristive coupled neural network to generate keys and dynamic S-boxes for a multi-layer XOR and substitution process. They implement the design on an Artix-7 FPGA and report about 0.8 Gbps throughput with strong statistical security results such as near-ideal entropy, near-zero pixel correlation, and good resistance to differential attacks.

In^23^, the authors propose using the NIST-standard lightweight authenticated-encryption algorithm ASCON for image encryption, implementing it in AEAD mode to produce cipher images with built-in authenticity protection. Rather than designing a new chaos-based method, they focus on demonstrating ASCON’s applicability to images and discuss a practical limitation: because ASCON processes data in a stateful sponge-based mode, ciphertext damage (e.g., noise/occlusion) can prevent successful recovery, which they suggest mitigating via pre-encryption row/column permutation.

While in^24^, the authors introduce GRTPHM, a multi-image encryption method that permutes pixels with a generalized rectangular transform (GRT) (suited to non-square images) and diffuses values via bit-level shuffling (BLRP) plus a 5-D hyperchaotic map (PHM) keyed using SHA-512. Their key novelty is an explicit inverse GRT (IGRT) that avoids the common “naïve inverse” failure on rectangular images and enables reliable decryption without knowing transform periodicity.

Furthermore, in^4^, the authors propose a novel Multiple Image Encryption (MIE) algorithm designed for satellite imagery. The method begins by augmenting multiple satellite images into a single composite, then encrypts each RGB channel through a four-stage process. First, additive confusion is applied using keys derived from a hyperchaotic memristor system and transformed via Singular Value Decomposition (SVD). Second, a counter-mode RC5 cipher with chaos-driven dynamic rotations is used to encrypt the bit-streams. Third, a Hill cipher is applied using matrices generated from a 6D hyperchaotic system, ensuring high security through invertibility modulo 256. Finally, a custom S-box generated via a chaos-enhanced Blum Blum Shub (BBS) algorithm introduces non-linearity. The result is a robust, real-time encryption scheme with high entropy, large key space ($2^{10524}$), low correlation, and strong resistance to differential, statistical, and brute-force attacks.

Clearly, recent advances have significantly improved the security and efficiency of satellite image encryption; however, several challenges remain. Many methods rely on complex chaotic systems or hardware implementations that may limit scalability or adaptability in real-world deployments. In addition, few approaches address key management, real-time processing under constrained resources, or resistance to emerging threats such as adversarial attacks. Future research should explore lightweight, adaptive encryption models, integration with AI-based threat detection, and standardized evaluation frameworks to ensure robustness across diverse operational scenarios.

The work in^25^ proposes a satellite image encryption pipeline driven by a 5D fractional‑order cosine memristive Hopfield neural network (5D‑FOCMHNN) that produces hyperchaotic sequences (with memory effects and multi-scroll/multistable behavior). For each RGB channel, it performs bit-level scrambling by sorting a coupled index sequence formed from multiple chaotic state streams, then applies dynamic DNA encoding where the chaotic output selects (per $8\times 8$ block) both the DNA rule and the DNA operation (XOR/add/sub/XNOR). Finally, it uses bidirectional cascade diffusion (forward then backward) in the DNA-symbol domain so small plaintext changes spread globally, yielding ciphertext with near-uniform statistics.

Positioning of the proposed chaos–chess pipeline. Although prior satellite-image encryption studies have combined chaotic dynamics with chess-inspired mechanisms, many chess-based designs employ the chess component primarily for pixel-level permutation/scrambling, followed by a conventional diffusion mask^16^. In contrast, the present work adopts a different coupling and stage ordering. Specifically, it introduces an invertible $2\times 2$ matrix mixing layer (mod 256) driven by a 6D fractional-order hyperchaotic system to provide lightweight algebraic confusion that is well-suited to parallel processing on large images^26^. It then applies per-channel, dynamically generated S-boxes instantiated online from a fractional-order hyperchaotic source, rather than relying on fixed or offline substitution tables, thereby strengthening key diversity across RGB channels^12,27,28^. Finally, instead of using chess motion only for spatial swapping, the Knight’s Tour is used as a traversal rule for block-wise bitstream diffusion on $8\times 8$ blocks, where the tour order governs XOR diffusion with hyperchaotic key-streams to amplify avalanche propagation^26,29^. Relatedly,^25^ demonstrates a complementary chaos-driven design that couples a fractional-order memristive hyperchaotic generator with dynamic DNA rule selection and bidirectional diffusion to achieve strong global sensitivity without relying on chess-derived traversals. This combination of local algebraic mixing, channel-specific dynamic substitution, and knight-guided bit-level diffusion yields a tightly coupled architecture that is structurally distinct from existing chaos+chess scrambling frameworks^16,26^.

A comparative summary of the reviewed methods, highlighting their adopted encryption techniques and application foci, is provided in Table 1 to facilitate further analysis and guide future developments.

**Table 1:** Comparison of satellite image encryption methods.

References	Encryption Technique	Application Focus	Chaos-Based	Real-Time Capable
^4^	Multi-stage: SVD + RC5 + Hill cipher + BBS S-box	Multiple image encryption	✓	✓
^12^	Dynamic S-box/P-box with JSMP map	General image encryption	✓	✗
^13^	Sin chaos + DNA transversion + ECC key hiding	Secure compression	✓	✗
^14^	AES (T-Box) + Hamming code	Satellite transmission reliability	✗	✓
^15^	Watermarking + chaotic encryption ($e\pi$ map)	Watermarked satellite images	✓	✗
^16^	Chess-based scrambling + 5D hyperchaos	Spatial scrambling for satellite images	✓	✓
^17^	Multi-layer chaos + Hill cipher + Kronecker XOR	Multi-layer encryption	✓	✗
^18^	7D chaos + RNA computing	DNA/RNA-inspired encryption	✓	✗
^19^	Galois ring-based Blowfish (reduced S-boxes)	Electro-optical satellite images	✗	✓
^20^	YOLOv7 + chaotic map + cross-channel diffusion	Selective encryption (maritime targets)	✓	✓
^21^	BSE + SHE + DH-optimized MSSMD	Secure classification pipeline	✓	✓
^22^	10D + 8D hyperchaos + memristive coupled neural network (MCNN); multi-layer XOR + dynamic S-box substitutions; FPGA (Artix-7) implementation	Multiple image encryption (high-throughput hardware / real-time)	✓	✓
^23^	ASCON (NIST lightweight AEAD) for image encryption evaluation	General image encryption (lightweight / real-time)	✗	✓
^24^	GRTPHM: generalized rectangular transform (GRT) permutation + BLRP diffusion + SHA-512-keyed 5D hyperchaotic PHM	Multiple (rectangular) image encryption	✓	✗
^25^	5D fractional-order cosine memristive Hopfield neural network (5D-FOCMHNN) keystream; multivariate bit-level scrambling; dynamic DNA encoding/operations; bidirectional diffusion	Satellite image encryption (robust scrambling–diffusion; noise/cropping resilience)	✓	✓

### Fractional-order hyperchaos

**Fig. 1:**
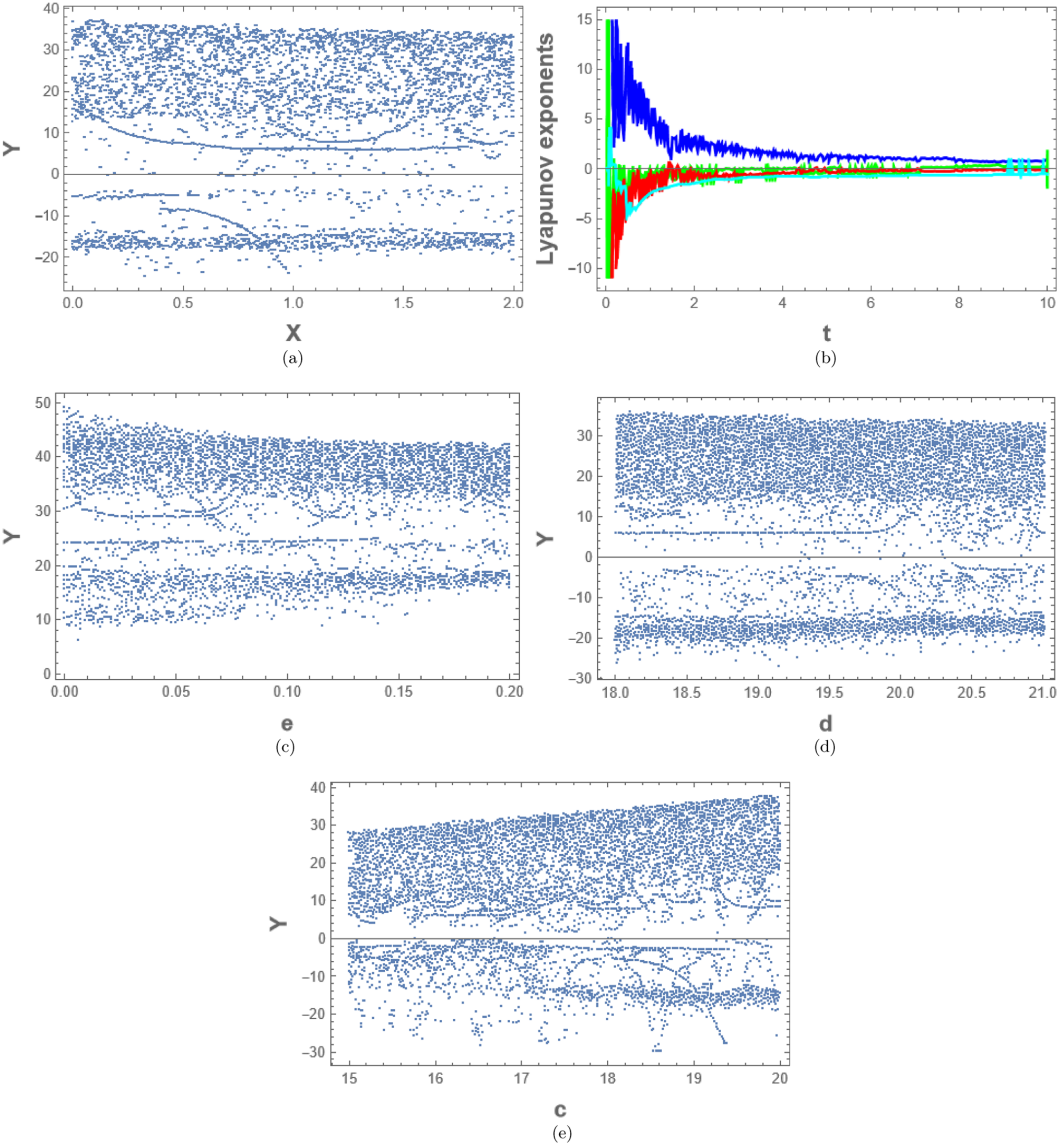
Bifurcation and Lyapunov exponents for 4D fractional order system.

**Fig. 2:**
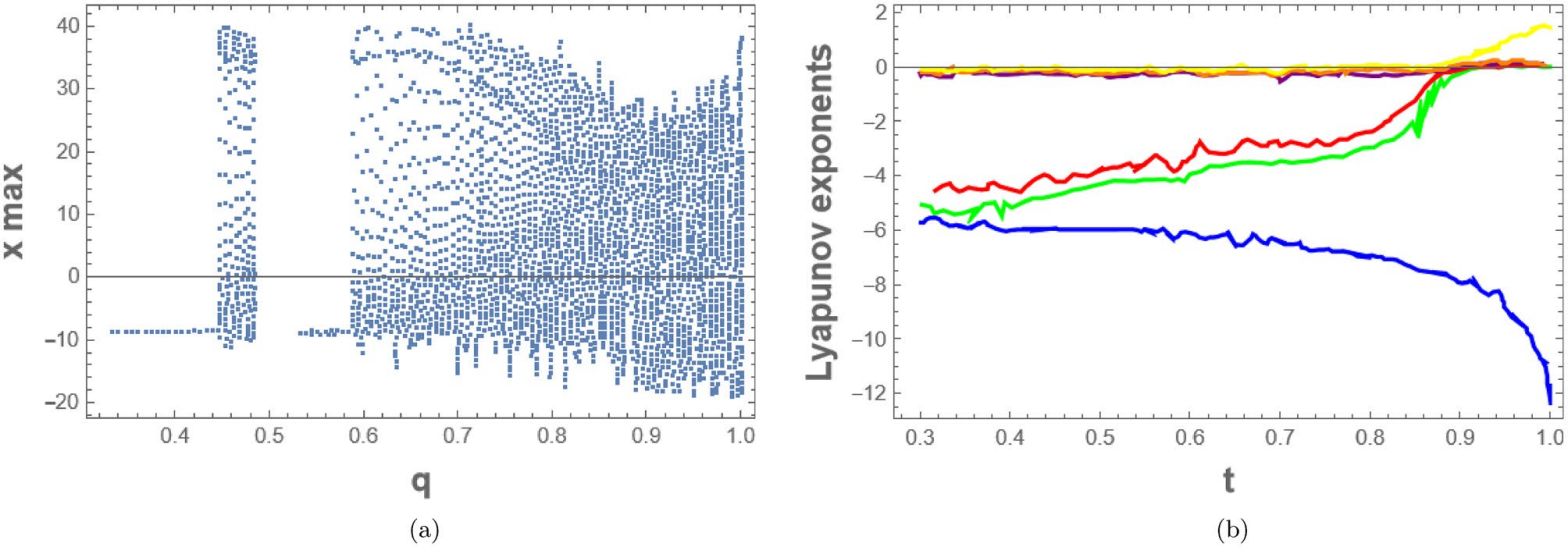
Bifurcation and Lyapunov exponents for 6D hyperchaotic fractional order system. (**a**) Bifurcation. (**b**) Lyapunov exponents.

Fractional-order (FO) chaotic systems generalize integer-order dynamics by introducing a nonlocal (history-dependent) operator, i.e., the state evolution depends not only on the current value but also on past states through the fractional derivative. From a cryptographic standpoint, this memory effect typically enriches the phase-space behavior and increases the dynamical complexity of the generated sequences, which is desirable when chaotic trajectories are used as key-stream sources. In the proposed framework, FO hyperchaos is not used as a generic “randomizer”; rather, it is tightly coupled to the cipher structure in three places. First, the 6D FO Vaidyanathan system is used to generate key-controlled invertible $2\times 2$ mixing matrices (mod 256), yielding lightweight algebraic confusion while preserving exact invertibility for decryption. Second, the 4D FO hyperchaotic system produces plaintext-sensitive sequences that are converted into online, per-channel S-box permutations, increasing key diversity and reducing reliance on fixed substitution tables. Third, the same FO-driven key-stream generation is employed during Knight’s Tour-guided bit-level diffusion so that small perturbations in the seed or plaintext propagate globally through the ciphertext. The selected parameter sets and fractional orders are taken from the cited sources and are verified to operate in a hyperchaotic regime via Lyapunov-exponent/bifurcation analysis (Figs. 1 and 2), thereby justifying FO hyperchaos as an appropriate key-stream backbone for the proposed satellite-image encryption pipeline.

## Mathematical constructs

### 4D hyperchaotic fractional order system

In^27^, the authors present a 4D fractional-order hyperchaotic system, and describe it mathematically by

1 $$\begin{aligned} \begin{aligned} \left\{ \begin{aligned}&D^{\alpha _4} x = \beta _1 y z,\\&D^{\alpha _4} y = \beta _2x-xz-\beta _3x,\\&D^{\alpha _4} z = \beta _4xy-xu,\\&D^{\alpha _4} u = x-\beta _5y+y,\\ \end{aligned} \right. \end{aligned} \end{aligned}$$

where initial conditions are $\{x,u,z,u\}=\{5, 1, 1, 1\}$ and parameters are $\{\beta _1,\beta _2,\beta _3,\beta _4,\beta _5\}=\{20,2,20,20,0.2\}$ with a Caputo fractional order $\alpha _4=0.98$. The system exhibits hyperchaotic behavior as mentioned in^27^, and is characterized by two Lyapunov exponents $\lambda _1=2.1253$ and $\lambda _2=0.00234$ with a Kaplan-Yorke dimension equal to 3.79783. Figure 1 displays various plots that verify the hyperchaotic behavior of the fractional order system in (1).

### 6D hyperchaotic fractional order system

In^26^, the authors present a 6D hyperchaotic Vaidyanathan fractional-order system, and describe it by

2 $$\begin{aligned} \begin{aligned} \left\{ \begin{aligned} D^{\alpha _6} x_1&= \beta _1 (x_2 - x_1) + x_4, \\ D^{\alpha _6} x_2&= \beta _2 x_1 - x_1 x_3 + x_4, \\ D^{\alpha _6} x_3&= x_1 x_2 - x_3 - x_4, \\ D^{\alpha _6} x_4&= -\beta _3 (x_1 + x_2) + x_5, \\ D^{\alpha _6} x_5&= -x_2 - \beta _4 x_4 + x_6, \\ D^{\alpha _6} x_6&= -\beta _5 (x_1 + x_5), \end{aligned} \right. \end{aligned} \end{aligned}$$

where $D^{\alpha _6}$ denotes the Caputo fractional derivative of order $\alpha _6=0.95$. The system is initialized with the conditions $\{x_1(0), x_2(0), x_3(0), x_4(0), x_5(0), x_6(0)\} = \{1, 1, 1, 1, 0, 0\}$. The parameters are chosen as $\{\beta _1, \beta _2, \beta _3, \beta _4, \beta _5\} = \{10, 76, 3, 0.2, 0.1\}$. For the specified parameter set, the system exhibits four positive Lyapunov exponents, indicating the presence of hyperchaotic dynamics, as discussed in ^26^, where the Kaplan-Yorke dimesion equals to 4.105. Figure 2 displays various plots that verify the hyperchaotic behavior of the fractional order system in (2).

### Knight’s tour

**Fig. 3:**
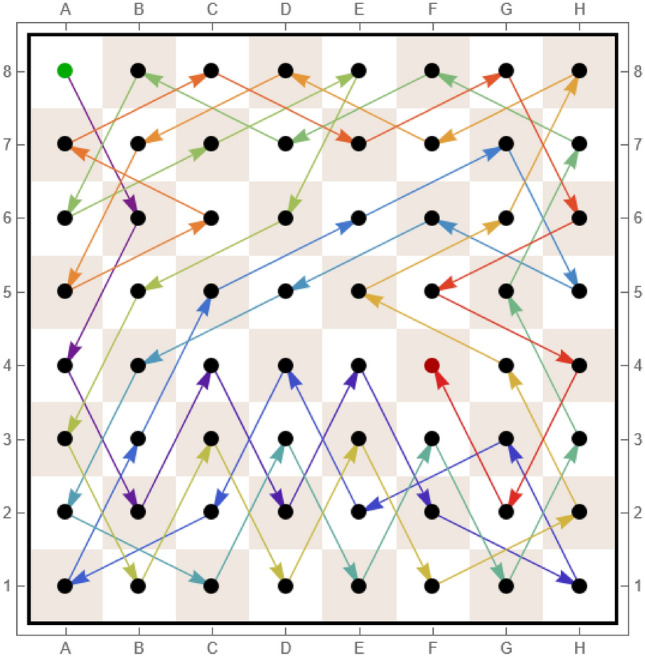
Knight’s tour path.

The Knight’s movement in chess is unique due to its asymmetric combination of horizontal and vertical traversals within a single move. A Knight’s Tour, illustrated in Fig. 3, represents a sequence of legal knight moves on a chessboard in which each square is visited exactly once. This problem is a specific instance of the Hamiltonian path problem and exists in two primary forms: the open tour, where the knight terminates on a different square, and the closed tour, where the knight returns to its initial position. For a standard $8\times 8$ chessboard, approximately 33.4 trillion distinct open Knight’s tours have been identified ^30^. Recent literature has shown the utilization of the Knight’s Tour in image encryption applications^29^.

In this work, the inherent nonlinearity of the Knight’s movement is utilized for image processing. The image is partitioned into multiple subblocks, with each subblock traversed according to a distinct Knight’s tour. This irregular traversal enhances the diffusion and confusion properties of the encryption process, thereby improving the overall cryptographic strength.

## Proposed algorithm

This section describes the encryption and decryption procedures for the proposed scheme.

### Encryption process

1. Stage 1:
  - Generate multiple plain images and augment them into a single image, giving $I_{A}$.
  - The image is separated into 3 different color channels (*RGB*), and each channel gets the value of pixels of the corresponding image, and the value of pixels is converted into *N* $2\times 2$ matrices giving $I_{[A,Red,2\times 2]}$, $I_{[A,Green,2\times 2]}$ and $I_{[A,Blue,2\times 2]}$.
  - Using $Seed_{6D}$ to solve the system in (2) to generate $Key_{6D}$, in a manner similar to that carried out in^28^. This key is converted into *N* $2\times 2$ matrices.
  - Then, matrix multiplication occurs between image pixel values and $Key_{6D}$ giving $I_{[A,Red,6D]}$, $I_{[A,Green,6D]}$ and $I_{[A,Blue,6D]}$.
    3 $$\begin{aligned} I_{[A,Red,6D]} = I_{[A,Red,2\times 2]} \times Key_{6D}, \end{aligned}$$
    4 $$\begin{aligned} I_{[A,Green,6D]} = I_{[A,Green,2\times 2]} \times Key_{6D}, \end{aligned}$$
    5 $$\begin{aligned} I_{[A,Blue,6D]} = I_{[A,Blue,2\times 2]} \times Key_{6D}. \end{aligned}$$
2. Stage 2:
  - Using Algorithm 1, $Seed_{4D}$ is used to solve the system in (1) to generate 3 different Sboxes as shown in Tables 2, 3 and 4 for different color channels giving $Sbox_{4D,Red}$, $Sbox_{4D,Green}$ and $Sbox_{4D,Blue}$.
  - Each pixel value resulting from stage 1 is converted into 1D bytes and invoked with their corresponding Sbox giving $I_{[A,Red,6D,Sbox]}$, $I_{[A,Green,6D,Sbox]}$ and $I_{[A,Blue,6D,Sbox]}$.
    6 $$\begin{aligned} I_{[A,Red,6D,Sbox]} = Sbox(I_{[A,Red,6D]}), \end{aligned}$$
    7 $$\begin{aligned} I_{[A,Green,6D,Sbox]} = Sbox(I_{[A,Green,6D]}), \end{aligned}$$
    8 $$\begin{aligned} I_{[A,Blue,6D,Sbox]} = Sbox(I_{[A,Blue,6D]}). \end{aligned}$$
3. Stage 3:
  - Converting each pixel values of every channel back into an image and then dividing it into $8\times 8$ image blocks to be similar to a chessboard and converting these pixel values into 1D bit-streams.
  - Using $Seed_{4D}$ to solve the system in (1) to generate 1D bit-streams giving $key_{4D}$.
  - Following Knight’s tour path in every image block, XORing occurs between image bit-streams and $Key_{4D}$ giving $I_{[A,Red,6D,Sbox,4D]}$, $I_{[A,Green,6D,Sbox,4D]}$ and $I_{[A,Blue,6D,Sbox,4D]}$.
    9 $$\begin{aligned} I_{[A,Red,6D,Sbox,4D]}=I_{[A,Red,6D,Sbox]} \oplus Key_{4D}, \end{aligned}$$
    10 $$\begin{aligned} I_{[A,Green,6D,Sbox,4D]}=I_{[A,Green,6D,Sbox]} \oplus Key_{4D}, \end{aligned}$$
    11 $$\begin{aligned} I_{[A,Blue,6D,Sbox,4D]}=I_{[A,Blue,6D,Sbox]} \oplus Key_{4D}. \end{aligned}$$
  - Finally, assemble every image block back and combine the *RGB* color channels into a single color channel that outputs the encrypted image $I'$.

**Fig. 4:**
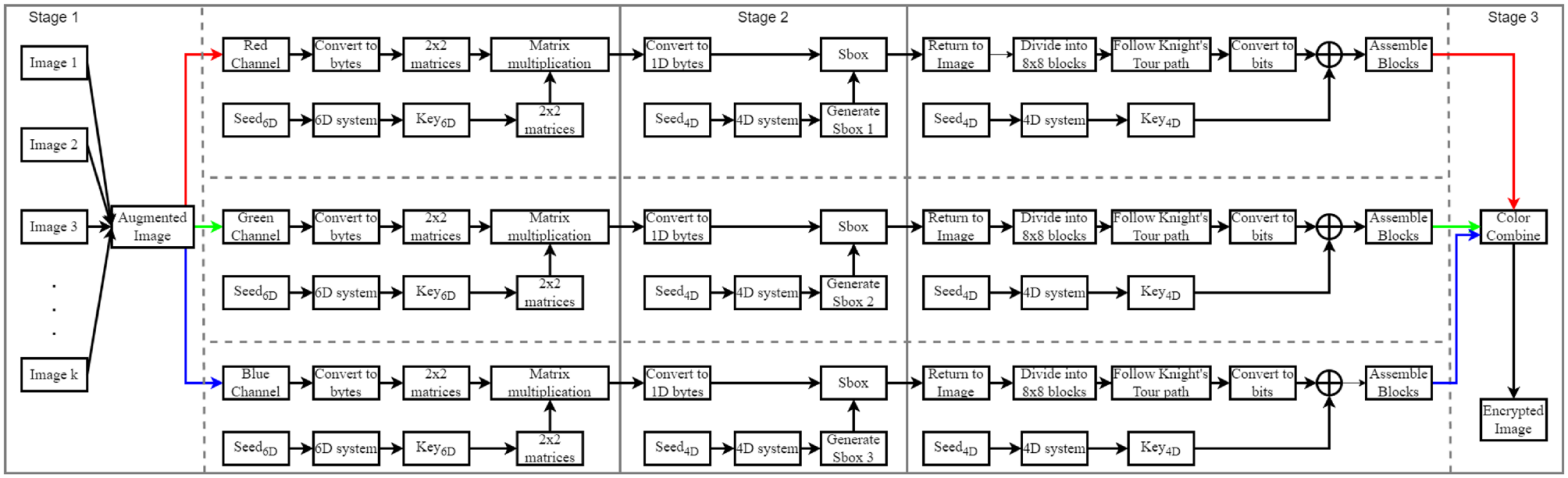
Encryption process flow chart.

The encryption procedure is visually represented as in Fig. 4.

**Table 2:** S-box generated from the 4D hyperchaotic fractional order system specified for the Red image channel.

12	172	120	135	22	216	129	23	251	186	40	237	131	6	168	200
0	13	32	29	84	92	90	180	79	116	196	72	35	46	98	27
56	20	156	205	244	125	89	68	4	153	21	219	143	101	226	36
154	187	39	162	236	9	185	223	11	3	106	65	109	228	73	113
30	142	95	25	195	15	100	147	181	37	1	209	49	189	190	171
203	26	17	145	85	240	133	118	220	62	211	136	87	119	127	238
108	227	19	54	150	96	34	31	166	74	121	53	177	57	174	122
104	235	194	76	14	66	222	169	110	43	157	140	152	208	198	252
241	253	199	221	248	2	61	44	88	163	102	97	134	234	112	50
42	5	188	103	137	144	33	184	45	146	232	176	111	7	99	141
246	233	24	179	115	161	158	155	224	126	170	77	204	165	69	255
192	132	123	214	10	107	47	242	124	159	247	52	51	230	75	55
78	191	148	201	210	151	71	83	59	64	160	8	213	175	193	254
105	67	114	197	207	28	81	91	38	41	231	138	93	130	178	117
48	128	218	225	206	164	60	16	202	215	245	80	173	217	212	183
229	139	239	249	58	82	182	18	250	149	63	86	94	70	167	243

**Table 3:** S-box generated from the 4D hyperchaotic fractional order system specified for the Green image channel.

33	23	112	36	209	202	1	65	238	26	104	147	62	185	4	205
41	50	6	14	179	106	108	113	210	10	199	158	35	168	165	206
28	173	170	157	42	145	248	121	123	153	39	227	254	20	177	143
38	72	73	15	132	7	13	12	92	180	74	225	60	96	190	186
78	197	3	164	189	90	195	63	77	251	87	174	222	200	61	85
135	57	84	149	128	252	161	105	115	127	64	126	218	160	120	8
118	137	156	151	19	52	249	31	178	133	142	70	198	98	182	139
241	55	176	130	203	58	134	69	122	220	68	235	101	221	216	83
159	208	201	144	59	114	21	138	124	236	211	53	192	80	155	214
56	237	91	67	79	110	188	253	103	213	9	129	32	66	30	223
181	34	234	47	255	166	217	194	107	230	76	125	247	245	116	102
204	81	187	82	27	162	100	24	95	18	97	167	94	175	219	250
207	140	117	150	226	48	46	88	93	212	37	169	183	231	136	43
109	184	152	232	51	224	131	11	229	2	233	240	244	75	242	243
246	193	89	99	154	146	54	239	45	25	0	86	22	172	196	191
17	163	29	40	148	16	44	171	228	215	111	5	71	119	141	49

**Table 4:** S-box generated from the 4D hyperchaotic fractional order system specified for the Blue image channel.

118	123	186	224	167	50	92	180	228	152	36	190	10	204	20	88
75	164	169	136	101	12	225	55	26	246	140	51	170	129	115	56
54	70	0	173	195	121	77	239	80	126	7	68	215	47	32	8
156	151	28	91	178	67	153	214	120	148	160	181	249	63	199	72
13	208	147	117	19	59	38	44	76	53	176	106	71	27	93	45
135	83	96	84	1	100	102	230	40	210	247	162	222	159	236	252
130	85	203	9	74	21	24	62	145	217	133	187	119	193	128	132
171	81	49	79	48	114	22	3	149	242	255	52	143	226	137	90
95	165	175	18	138	221	216	66	206	39	131	205	33	196	57	201
41	141	86	213	89	183	34	191	244	110	122	87	69	157	29	202
251	161	142	200	94	98	16	163	179	134	97	105	223	2	31	30
78	82	229	60	144	237	146	211	35	104	234	65	108	112	177	198
197	248	245	168	46	155	109	254	185	139	207	14	58	154	243	231
23	212	253	17	11	227	189	37	6	103	240	233	232	172	184	43
61	219	113	218	127	5	209	192	182	15	166	42	194	4	73	150
124	235	107	220	25	99	188	241	116	250	174	125	111	158	238	64

### Decryption process

1. Stage 3:
  - The encrypted image $I'$ is separated into *RGB* color channels.
  - Each color channel is divided into $8\times 8$ blocks, similar to a chessboard. The pixel values of each block are converted into a 1D bit-streams.
  - Using the same $Seed_{4D}$ is reused to solve the system in (1) to regenerate the bit-streams key $Key_{4D}$.
  - XOR operation is performed using the Knight’s Tour path giving $I_{[A,Red,6D,Sbox]}$, $I_{[A,Green,6D,Sbox]}$ and $I_{[A,Blue,6D,Sbox]}$.
    12 $$\begin{aligned} I_{[A,Red,6D,Sbox]} = I_{[A,Red,6D,Sbox,4D]} \oplus Key_{4D}, \end{aligned}$$
    13 $$\begin{aligned} I_{[A,Green,6D,Sbox]} = I_{[A,Green,6D,Sbox,4D]} \oplus Key_{4D}, \end{aligned}$$
    14 $$\begin{aligned} I_{[A,Blue,6D,Sbox]} = I_{[A,Blue,6D,Sbox,4D]} \oplus Key_{4D}. \end{aligned}$$
2. Stage 2:
  - The decrypted bit-streamss are then converted back into 1D bytes.
  - Using the same $Seed_{4D}$ is again used to generate the S-boxes for each color channel: $Seed_{4D,Red}$, $Seed_{4D,Green}$, and $Seed_{4D,Blue}$.
  - An inverse S-box is applied to the pixel values giving $I_{[A,Red,6D]}$, $I_{[A,Green,6D]}$ and $I_{[A,blue,6D]}$.
    15 $$\begin{aligned} I_{[A,Red,6D]} = Sbox^{-1}(I_{[A,Red,6D,Sbox]}), \end{aligned}$$
    16 $$\begin{aligned} I_{[A,Green,6D]} = Sbox^{-1}(I_{[A,Green,6D,Sbox]}), \end{aligned}$$
    17 $$\begin{aligned} I_{[A,Blue,6D]} = Sbox^{-1}(I_{[A,Blue,6D,Sbox]}). \end{aligned}$$
3. Stage 1:
  - The same $Seed_{6D}$ is reused to regenerate the key matrix $Key_{6D}$ by solving the system in (2), in a manner similar to that carried out in^28^. The key is reconstructed as *N* $2\times 2$ matrices.
  - Matrix inversion is performed on each $Key_{6D}$ matrix to obtain $Key_{6D}^{-1}$.
  - Each of the color matrices is then multiplied by the inverse key to recover the original $2\times 2$ pixel value matrices:
    18 $$\begin{aligned} I_{[A,Red,2\times 2]} = I_{[A,Red,6D]} \times Key_{6D}^{-1}, \end{aligned}$$
    19 $$\begin{aligned} I_{[A,Green,2\times 2]} = I_{[A,Green,6D]} \times Key_{6D}^{-1}, \end{aligned}$$
    20 $$\begin{aligned} I_{[A,Blue,2\times 2]} = I_{[A,Blue,6D]} \times Key_{6D}^{-1}. \end{aligned}$$
  - Finally, the *RGB* channels are merged to reconstruct the original image $I_{A}$.

**Fig. 5:**
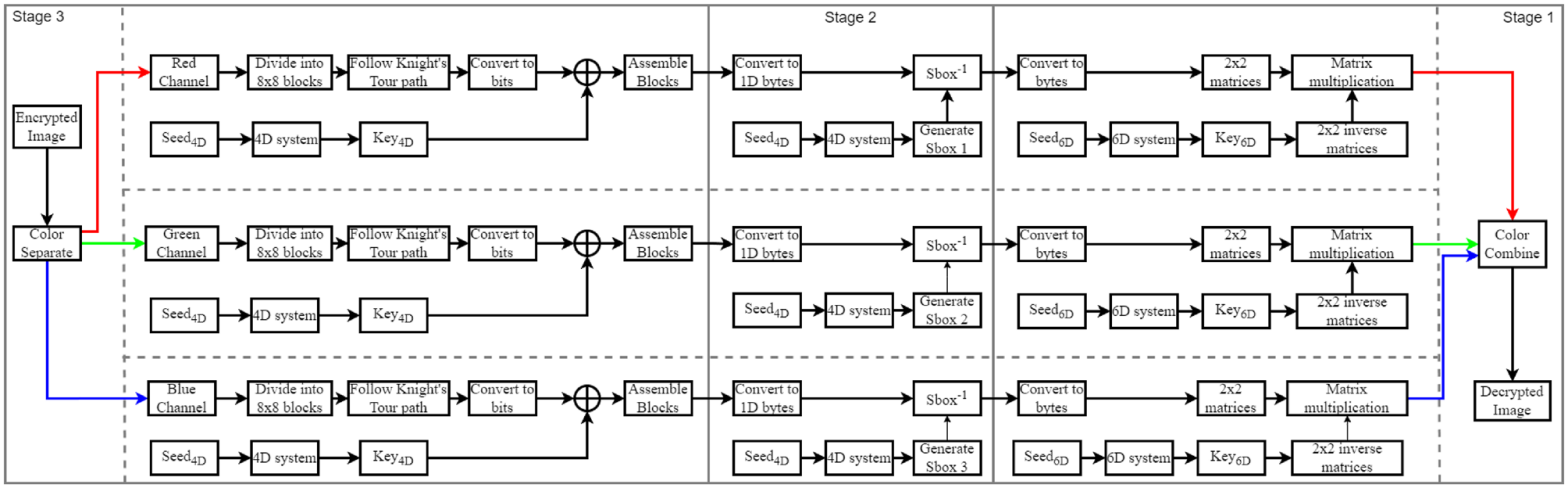
Decryption process flow chart.

The decryption procedure is visually represented as in Fig. 5.

### Practical efficiency and scalability

The proposed cipher is designed to remain practical for high-resolution satellite imagery by relying on constant-time operations per pixel and by using block-local processing that enables parallel execution. For an $M\times N$ color image, Stage 1 performs a modular $2\times 2$ mixing per 4 pixels (constant arithmetic cost per block), Stage 2 applies a single S-box lookup per byte, and Stage 3 performs XOR-based diffusion over fixed-size $8\times 8$ blocks following a precomputed Knight’s Tour order. Hence, the overall encryption/decryption complexity scales approximately linearly with the number of processed pixels, i.e., *O*(*MN*), which matches the empirical scaling trend, as will be shown in Subsect. 5.7. Importantly, the pipeline is naturally parallelizable: the $2\times 2$ matrix mixing and per-byte substitution can be executed independently across blocks and across RGB channels, and the diffusion stage can be parallelized across $8\times 8$ blocks. The fractional-order hyperchaotic generators are used to produce key stream material and dynamic S-boxes once per image/session; the subsequent encryption passes consist of lightweight integer operations (mod 256 arithmetic, table lookup, and XOR), making the scheme suitable for SIMD/GPU acceleration and FPGA implementations in bandwidth-constrained remote-sensing pipelines^22,31,32^.

## Performance evaluation

This section presents the results of the performance evaluation of the proposed algorithm. Table 5 provides the mathematical expressions of the utilized metrics. Test images are sourced from the SIPI website^33^ and the satellite images from^34^. All images were resized to $256 \times 256$, unless otherwise stated. The algorithm is implemented on a machine equipped with an AMD^®^ Ryzen 5 5600H processor and 16 GB of RAM.

Finite-precision arithmetic is known to degrade digitally implemented chaotic dynamics (e.g., by inducing short cycles or reduced complexity). Accordingly, the digital implementation assumptions are stated explicitly, and a numeric precision on the order of $10^{-16}$ is used during key-material generation from the fractional-order hyperchaotic systems; the encryption core is then executed primarily in the integer domain via mod-256 $2\times 2$ mixing, S-box lookups, and XOR-based diffusion. To reduce potential degradation, the generated real-valued sequences are discretized/whitened into bytes/bits before use, and a complete $0-255$ permutation is enforced during S-box construction (Algorithm 1) to limit bias arising from repeated or correlated samples.

In accordance with Kerckhoffs’ principle^35^, the proposed scheme is evaluated assuming the algorithm is public and only the secret key remains unknown, under adversary models that include ciphertext-only, known-plaintext, and chosen-plaintext access. The security objectives are practical confidentiality for remote-sensing imagery, with emphasis on resistance to statistical attacks (histogram and correlation analysis), differential attacks (NPCR/UACI), and strong key sensitivity.

### Statistical analysis

The results presented in Table 6 validate the efficacy of the proposed encryption scheme. The high Mean Squared Error (MSE) and low Peak Signal-to-Noise Ratio (PSNR) values confirm significant distortions induced by the encryption process. This is further supported by the Mean Absolute Error (MAE), which validates considerable pixel-level alterations. The entropy of the encrypted images ($H_E$) is approximately 8, which is near the ideal value, indicating a high degree of randomness and affirming the cryptographic robustness of the scheme. Furthermore, the pixel correlation coefficient (PCC) analysis, detailed in Table 7 demonstrates that original images maintain strong correlations between adjacent pixels. In contrast, the encrypted images exhibit correlations near zero, confirming the effective randomization of pixel relationships. Collectively, these performance metrics demonstrate the capability of the proposed image encryption scheme to effectively disrupt statistical and structural dependencies, thereby ensuring a high level of security.

**Table 5:** Mathematical expressions of the performance evaluation metrics.

Metric	Descriptive equation(s)
PCC	$\rho (x,y)= \frac{cov(x,y)}{\sqrt{\sigma (x)}\sqrt{\sigma (y)}},$ where $$\begin{gathered} cov(x,y) = \frac{1}{N}\sum\limits_{{i = 1}}^{N} {(x_{i} - \mu (x))(y_{i} - \mu (y))} , \hfill \\ \sigma (x) = \frac{1}{N}\sum\limits_{{i = 1}}^{N} {(x_{i} - \mu (x))^{2} } , \hfill \\ \mu (x) = \frac{1}{N}\sum\limits_{{i = 1}}^{N} {(x_{i} )} . \hfill \\ \end{gathered}$$
MSE	$MSE = \frac{\sum _{i=0}^{M-1} \sum _{j=0}^{N-1} (I_{i,j} - I'_{i,j})^2}{M \times N}$
PSNR	$PSNR = 10 \log _{10} \left( \frac{I_{max}^2}{MSE}\right) , \quad I_{max} = 255$
Entropy	$H(m) = \sum _{i=1}^M p(m_i) \log _2 \frac{1}{p(m_i)}$
NPCR	$NPCR = \frac{1}{MN} \sum _{i=1}^{M}\sum _{j=1}^{N} D(i,j) \times 100\%$ where $$\begin{gathered} D(i,j) \hfill = \left\{ {\begin{array}{*{20}l} {1,} \hfill & {{\text{if }}I_{e}^{1} (i,j) \ne I_{e}^{2} (i,j)} \hfill \\ {0,} \hfill & {{\mathrm{otherwise}}.} \hfill \\ \end{array} } \right. \hfill \\ \end{gathered}$$
UACI	$UACI = \frac{1}{MN} \sum _{i=1}^{M}\sum _{j=1}^{N} \frac{|I_e^1(i,j) - I_e^2(i,j)|}{255} \times 100\%$
MAE	$MAE=\frac{1}{M\times N}\sum _{i=0}^{M-1}\sum _{j=0}^{N-1}|P_{(i,j)}-E_{(i,j)} |,$
DFT	$F(k,l)=\sum _{x=0}^{N-1} \sum _{y=0}^{N-1} f(x,y) e^{-j 2\pi (\frac{k x}{N}+\frac{l y}{N})}.$

**Table 6:** Statistical analysis on various images.

Images	MSE	PSNR	Entropy	NPCR	UACI	MAE
Mandrill	8254.93	8.96367	7.99893	99.6206	29.3499	74.8422
Peppers	10077.7	8.0972	7.99903	99.6002	32.1668	82.0252
House	8357.51	8.91003	7.99917	99.6099	29.5363	75.3175
Sat. Image 1	10205.2	8.04257	7.99881	99.6134	32.3443	82.478
Sat. Image 2	8853.8	8.6595	7.99894	99.6099	30.2918	77.2441
Sat. Image 3	9976.91	8.14084	7.99913	99.649	32.0306	81.6779
Average	9287.675	8.4689	7.999001	99.6171	30.9532	78.9274

**Table 7:** Correlation coefficient of various images.

	Plain Image			Encrypted Image		
Images	H	D	V	H	D	V
Mandrill	0.848778	0.750624	0.79088	0.00539006	$-0.00572371$	$-0.00275938$
Peppers	0.959422	0.930426	0.966795	$-0.00477652$	0.00384722	0.00625127
House	0.978232	0.936044	0.952926	0.0000995145	$-0.000907099$	0.00320291
Sat. Image 1	0.968346	0.933208	0.963178	0.000045445	$-0.000357486$	0.00286339
Sat. Image 2	0.902237	0.846318	0.922587	0.00407169	0.00210273	$-0.00254914$
Sat. Image 3	0.906315	0.872142	0.927558	0.00498931	0.00116956	0.00257499

**Table 8:** Comparative analysis with various schemes.

Scheme	PSNR	Entropy	NPCR	UACI	Key space	Time [s]	Machine specifications	Normalized Time [s]
Proposed	8.4689	7.999001	99.6171	30.9532	$2^{3827}$	0.851602	AMD^®^ Ryzen^TM^ 5600H, 3.3 GHz, 16 GB	2.8102866
^4^	8.6538	7.99907	99.6076	30.6095	$2^{10524}$	0.230717	Intel^®^ i9 @ 2.9 GHz, 32 GB	0.6690793
^14^	−	7.9998	99.81519	33.34718	−	−	Virtex-4QV XQR4VSX55	−
^16^	−	7.9981	99.6308	33.4552	$2^{647}$	1.2445	−	−
^17^	8.76267	7.9982	99.6038	33.5333	$2^{160}$	−	−	−
^18^	−	7.9985	99.61	33.43	−	0.983	−	−
^20^	−	7.9974	99.6023	33.4535	$2^{180}$	0.4610	Intel^®^ Core(TM) i5-12500H, 2.50 GHz, 16 GB	23.05
^22^	8.29252	7.99906	99.6173	31.6394	$2^{3454}$	0.0838975	Intel^®^ Core(TM) i5-10510U, 1.8 GHz, 16 GB	0.1510155
^23^	8.1262	7.9988	99.6267	33.5581	−	−	−	−
^24^	15.41	7.999616	99.6077	33.47344	$2.8639\times 10^{250}$	0.3402	CPU 2.80 GHz, 16 GB	0.95256

### Visual and histogram analysis

**Fig. 6:**
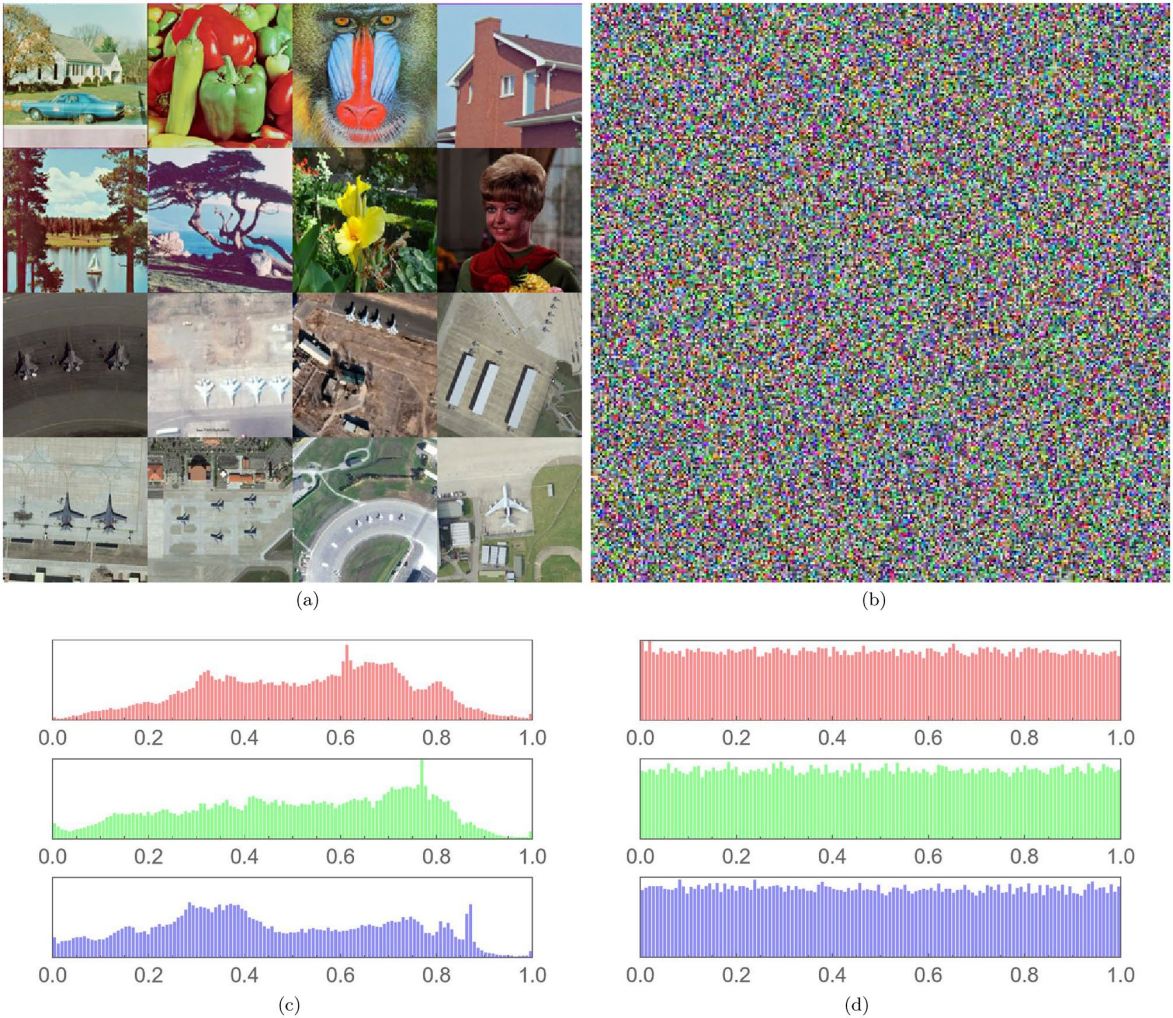
Images and histograms. (**a**) Plain augmented image. (**b**) Encrypted augmented image. (**c**) Augmented plain histogram and (**d**) Augmented encrypted histogram.

**Fig. 7:**
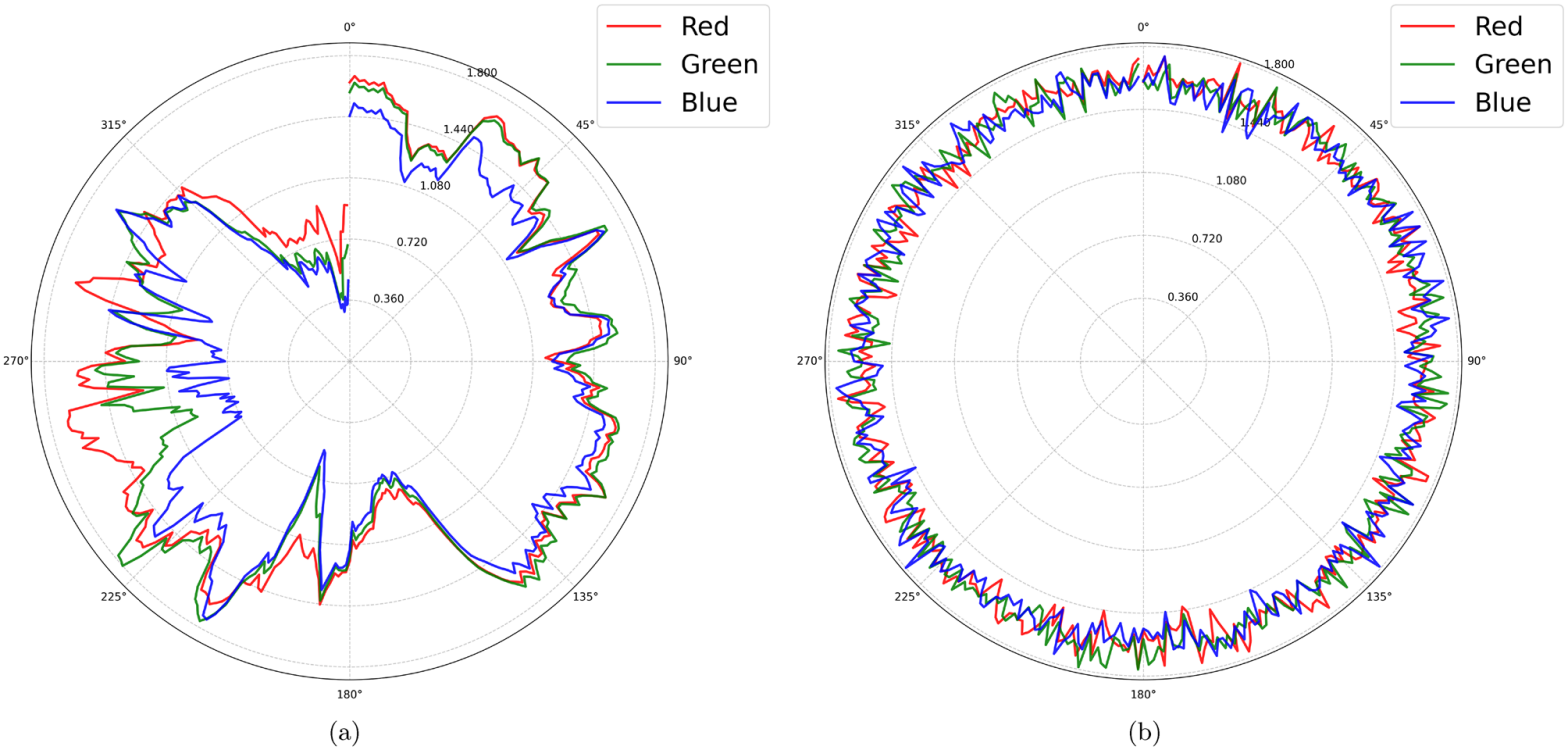
Polar histogram analysis. (**a**) Polar histogram for plain image. (**b**) Polar histogram for encrypted image.

Visual analysis confirms the efficacy of the proposed algorithm, as shown in Fig. 6 for both satellite and non-satellite images. The encrypted output exhibits a noise-like appearance, ensuring no discernible information from the original image is retained, while the decrypted images are perfectly reconstructed without any distortion or artifacts, verifying lossless recovery. Furthermore, histogram analysis, also in Fig. 6, demonstrates that the distinct patterns of the plain images are transformed into a uniform distribution in the cipher images, thereby effectively hiding original content and mitigating statistical information leakage. The efficacy of the encryption algorithm is evaluated through polar histogram analysis, as depicted in Fig. 7. The polar histogram for the original plaintext image, shown in Fig. 1a, exhibits significant fluctuations and distinct peaks, revealing the non-uniform distribution of pixel intensities and the inherent statistical patterns within the uncompressed data. In contrast, the polar histogram for the ciphertext image, presented in Fig. 1b, demonstrates a substantially flatter and more uniform distribution. This absence of prominent features and the random-like distribution of intensity values confirm that the encryption process successfully diffuses and confuses the pixel information. The results verify that the proposed method effectively mitigates statistical attacks by producing an encrypted output that bears no resemblance to the statistical profile of the original image. The consistent performance across diverse image types and the perfect histogram match after decryption validate the algorithm’s robustness and its ability to ensure security irrespective of input characteristics (Table 8).

**Algorithm 1:**
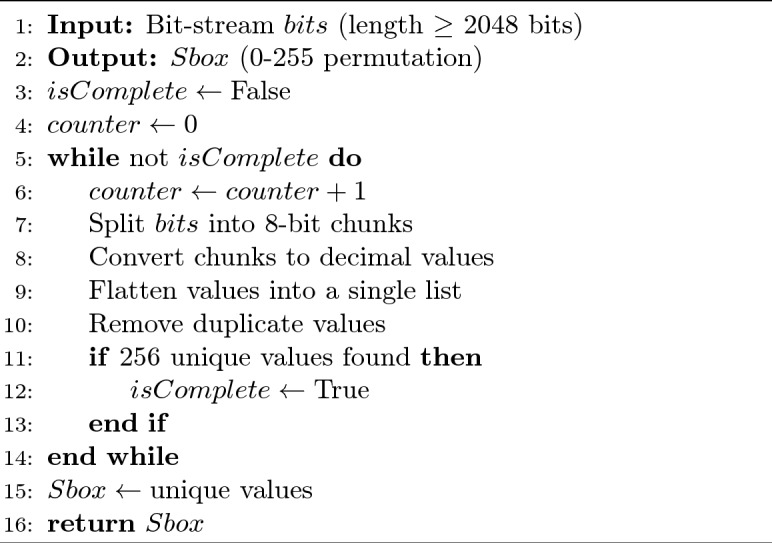
S-box Construction from Bit-stream (adapted form^28^)

###  S-box performance analysis

**Fig. 8:**
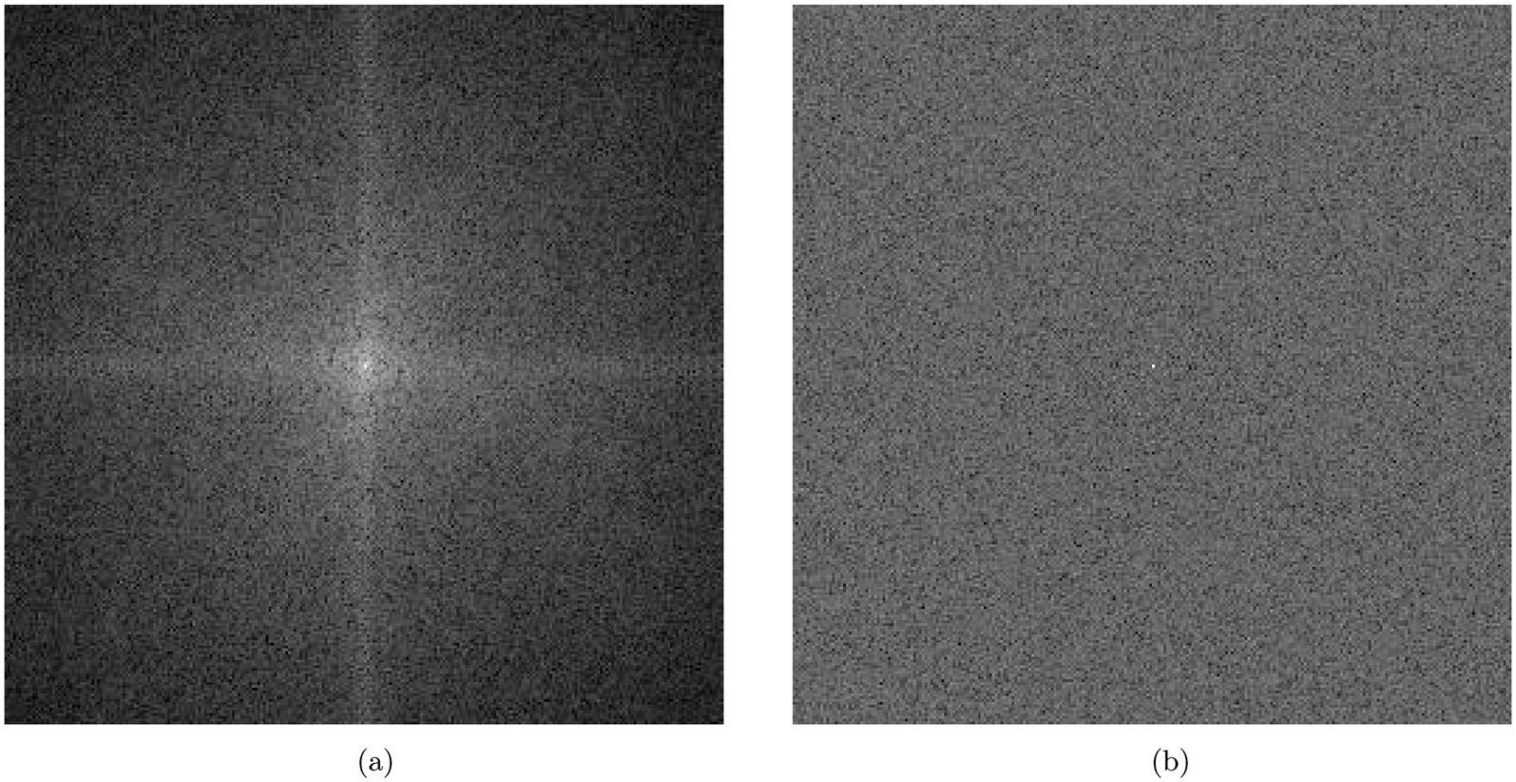
DFT as applied to plain and encrypted augmented image. (**a**) DFT of plain image. (**b**) DFT of encrypted image.

Substitution boxes (S-boxes) are fundamental non-linear components in cryptographic systems, crucial for ensuring security. The cryptographic strength of an S-box is evaluated through several critical criteria:

1. *Non-linearity (NL)* This metric measures the minimum Hamming distance between the S-box’s Boolean functions and the set of all affine functions^36^. A high NL value indicates strong resistance to linear cryptanalysis by preventing affine approximations.
2. *Linear Approximation Probability (LAP)* LAP quantifies the maximum bias in linear relations between input and output bits^37^. A lower LAP value denotes enhanced resistance to linear attacks by minimizing predictable correlations.
3. *Differential Approximation Probability (DAP)* DAP evaluates the probability that a given input difference leads to a specific output difference^38^. A secure S-box achieves a low DAP value, ensuring robustness against differential cryptanalysis.
4. *Bit Independence Criterion (BIC)* BIC assesses the statistical independence between pairs of output bits when a single input bit is complemented^39^. An ideal S-box satisfies BIC by ensuring output bits change independently.
5. *Strict Avalanche Criterion (SAC)* SAC requires that complementing a single input bit results in each output bit changing with a probability of 0.5^39^. This ensures a strong avalanche effect and enhances diffusion (Fig. 8).

Table 9 indicates the proposed S-box design. The experimental results indicate that the proposed S-box meets or exceeds the security performance of state-of-the-art designs, confirming its suitability for applications requiring high security, such as satellite image encryption.

**Table 9:** Comparison among the proposed S-boxes and those in the literature.

S-Box	NL	SAC	BIC	LAP	DAP
Ideal Values	112	0.5	112	0.0625	0.015625
Proposed S-box 1	108	0.506592	108	0.0781	0.015625
Proposed S-box 2	108	0.507812	108	0.0781	0.015625
Proposed S-box 3	108	0.506104	108	0.0781	0.015625
^3^	108	0.49821	105.3	0.0885	0.015625
^4^	108	0.501546	105.33	0.0989	0.015625
^7^	108.66	0.501465	108	0.0781	0.015625
^27^	112	0.5725	103.36	0.1406	−

### Further attacks

**Fig. 9:**
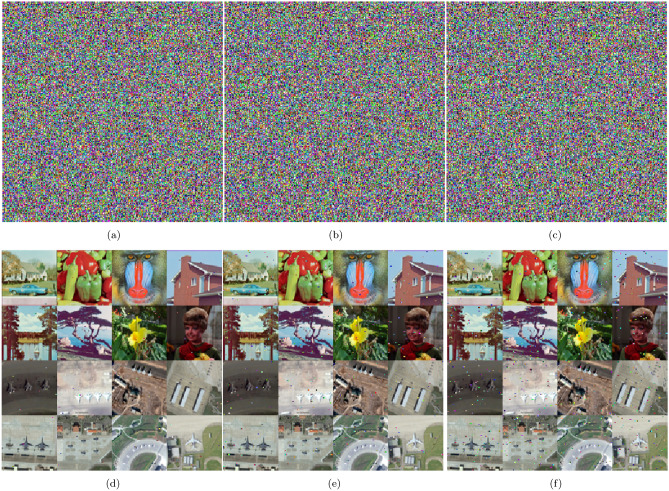
A visual representation of Salt and Peppers attack with different percentages. (**a**) 1%. (**b**) 5%. (**c**) 10%. (**d**) 1% and (**e**) 5%. (**f**) 10%.

**Fig. 10:**
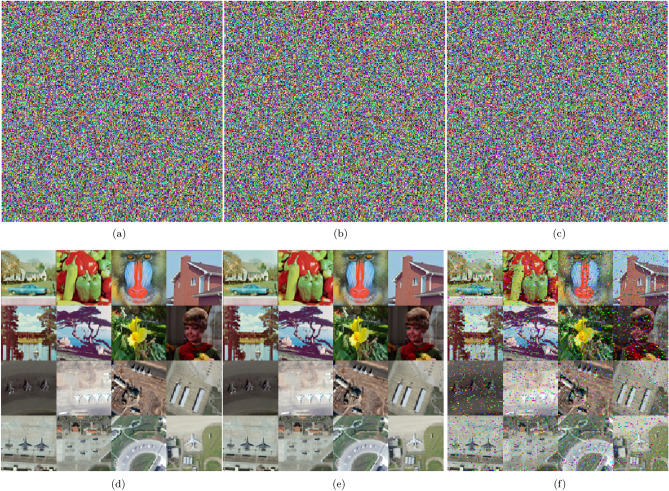
A visual representation of Gaussian noise attack with different intensities. (**a**) σ = 0.0001. (**b**) σ = 0.0005. (**c**) σ = 0.001. (**d**) σ = 0.0001. (**e**) σ = 0.0005 and (**f**) σ = 0.001.

**Fig. 11:**
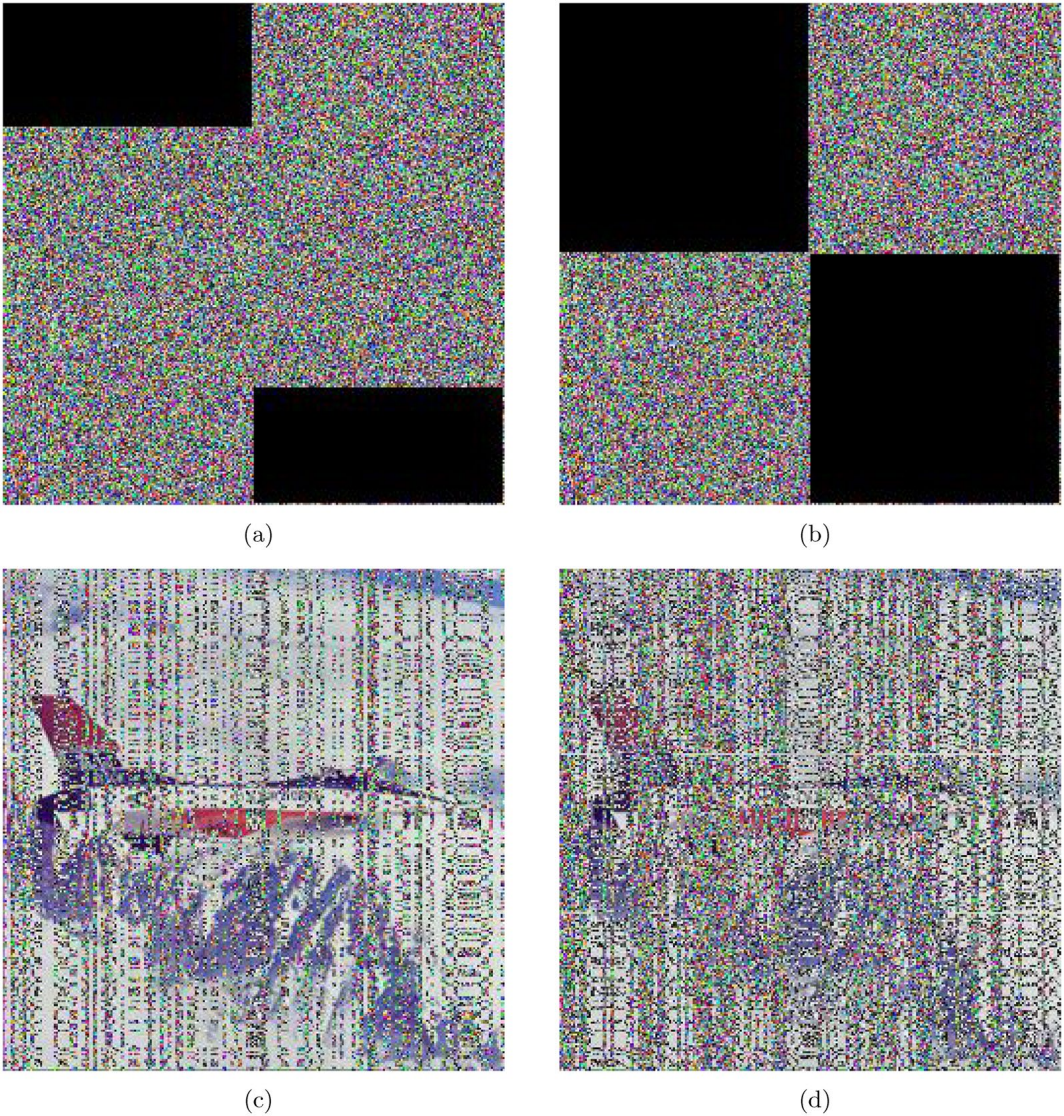
A visual representation of occlusion attacks with different occluded areas. (**a**) 25%. (**b**) 50%. (**c**) 25% and (**d**) 50%.

This section evaluates the robustness of the proposed encryption algorithm under various attacks and adverse conditions, as illustrated in Fig. 9, 10, and 11.

Figure 9 demonstrates the algorithm’s performance against salt-and-pepper noise with varying noise densities of $1\%$, $5\%$, and $10\%$. The encrypted images exhibit a high degree of randomness, with no discernible patterns or information leakage, confirming the algorithm’s ability to preserve data confidentiality even under severe noise interference.

Figure 10 depicts the algorithm’s resilience to Gaussian noise with standard deviations of $\sigma = 0.0001$, 0.0005, and 0.001. The results indicate that the encrypted images retain their statistical uniformity and visual in detectability across all noise levels, underscoring the scheme’s robustness against typical noise encountered during transmission and storage.

Figure 11 demonstrates the algorithm’s performance against occlusion attacks with varying occluded areas of $25\%$ and $50\%$. The results indicate that in the first attack case, the recovered images maintain a recognizable visual appearance. In the second attack case, despite the substantially increased attack strength, meaningful visual information remains identifiable in the decrypted images, highlighting the robustness of the proposed scheme against strong noise interference.

Figures ( 12 and 13) evaluates the robustness of the proposed framework against chosen-plaintext attacks; experiments are conducted using highly structured and user-defined input images, such as constant-valued images. In a CPA scenario, the attacker has the ability to select arbitrary plaintext inputs and analyze the corresponding outputs in an attempt to infer the secret key or underlying transformation. The selected images are processed through the full encryption pipeline. The resulting encrypted outputs exhibit noise-like characteristics with no observable structural patterns or correlations with the chosen inputs. Despite the simplicity and predictability of the plaintext images, the corresponding outputs remain statistically uniform and visually random.

Furthermore, Table 10 presents a numerical comparison of occlusion attack results for multiple test images against existing schemes reported in the literature. The proposed encryption method yields higher MSE values and lower PSNR under occlusion attacks, indicating enhanced security performance at the expense of reduced robustness. Although this behavior limits image recoverability under partial data loss, it strengthens confidentiality, thereby emphasizing the inherent trade-off between security and robustness.

Collectively, these results validate the proposed algorithm’s strong resistance to occlusion and noise-based attacks, affirming its applicability in high-security domains such as satellite and remote-sensing imaging, where maintaining both data integrity and confidentiality is critical.

**Fig. 12:**
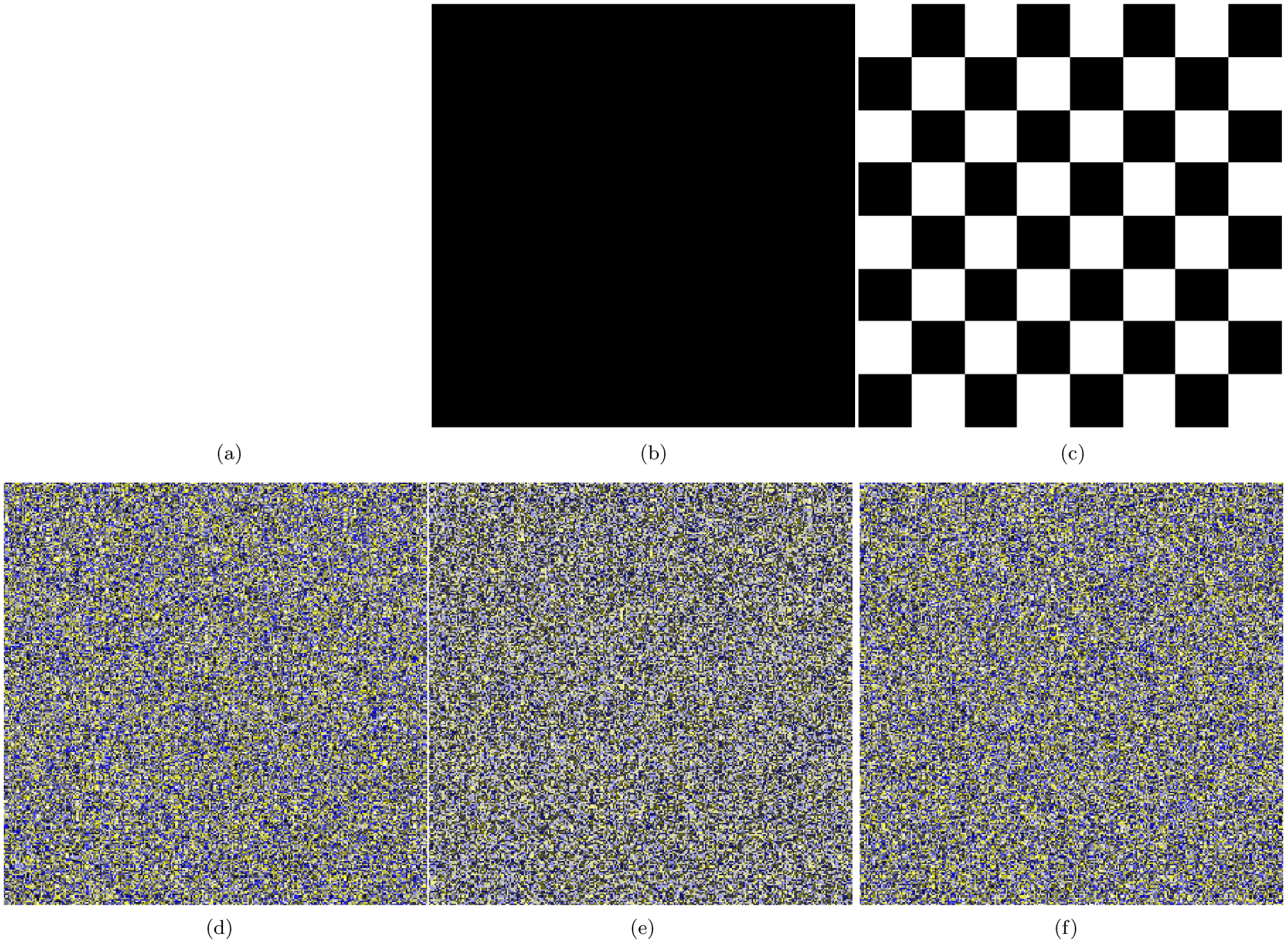
A visual representation of chosen-plaintext attacks. (**a**) Plain white image. (**b**) Plain black image. (**c**) Plain black and white image. (**d**) Encrypted white image. (**e**) Encrypted black image and (**f**) Encrypted black and white image.

**Fig. 13:**
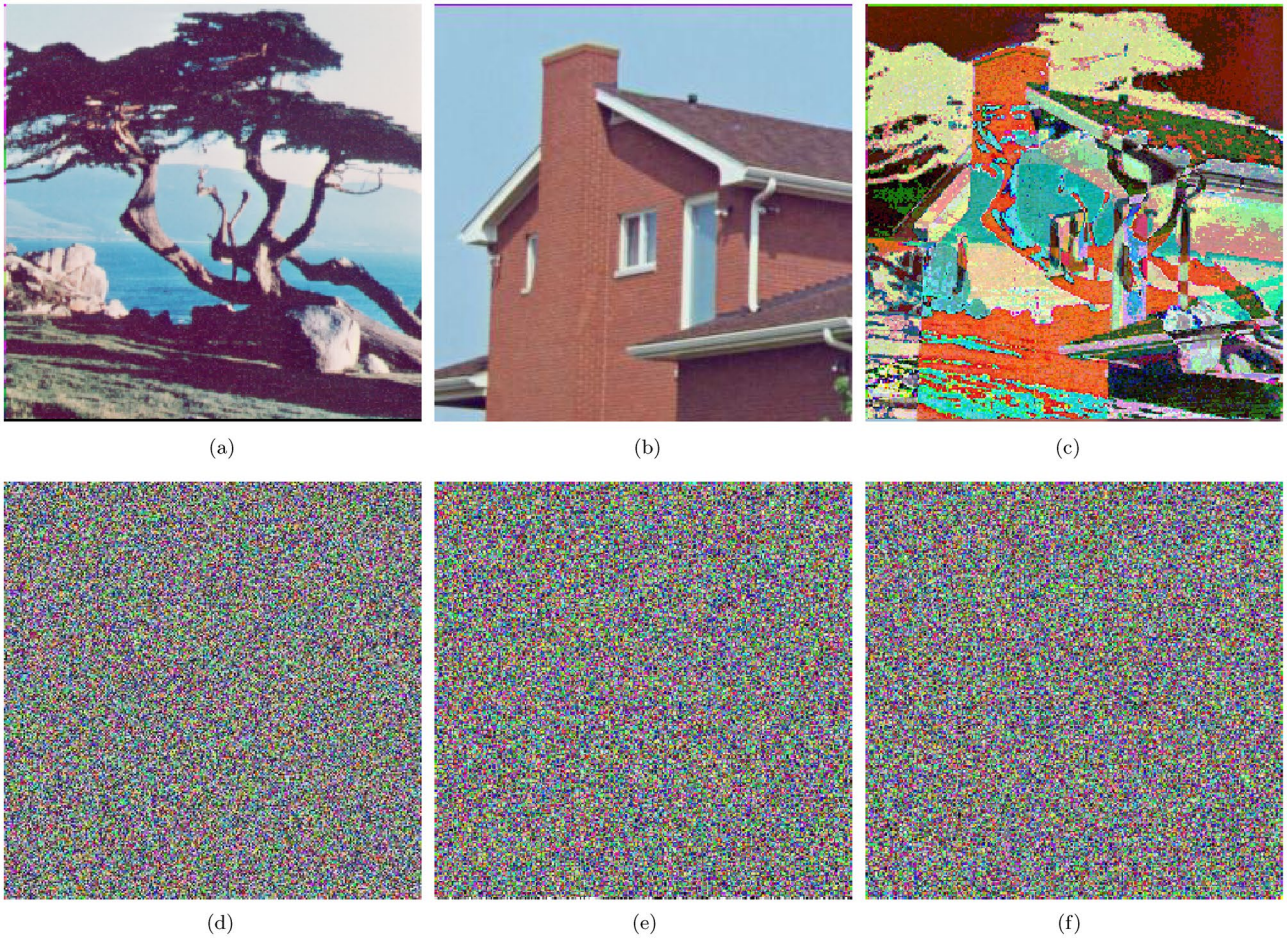
Another visual representation of chosen-plaintext attacks. (**a**) Tree plain. (**b**) House plain. (**c**) Encrypted image between two plain. (**d**) House Encrypted. (**e**) Tree encrypted and (**f**) Encrypted image between two encrypted.

**Fig. 14:**
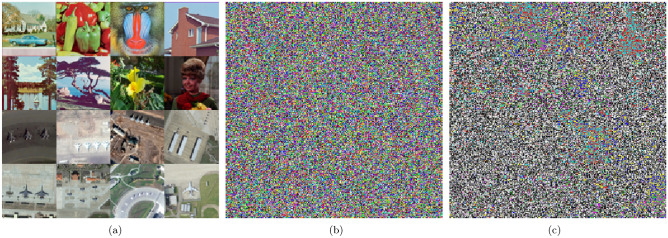
Ablation test by removing stages from the proposed scheme. (**a**) Plain Augmented. (**b**) One Stage removed and (**c**) Two Stages removed.

**Table 10:** Comparison of noise performance analysis with recent literature.

Noise Type	Ratio	Metric	Proposed			^40^			^41^			^42^		
			Mandrill	Peppers	F16	Mandrill	Peppers	F16	Mandrill	Peppers	F16	Mandrill	Peppers	F16
Occlusion Attack	1/4	PSNR	7.5117	7.39631	6.31577	10.425	10.537	10.45	7.677	7.283	6.176	6.35675	7.5066	6.35675
		MSE	11532.2	11842.7	15188.1	6508.43	6570.4	6721.35	11101.20	12155.50	15685.60	15045.4	11545.7	15045.4
	1/2	PSNR	6.28049	6.31442	5.59077	12.558	12.151	12.116	6.493	6.328	5.183	7.78877	7.82905	6.51838
		MSE	15312	15192.8	17947.5	478.21	4856.1	4881.2	14580.2	15144.3	19715	10819.4	10719.5	14495.8
Gaussian Noise	0.0001	PSNR	8.93967	8.10882	7.98361	7.861	7.84	7.562	8.92697	8.10321	7.98535	–	–	–
		MSE	8300.67	10050.7	10344.7	9706.5	9970.2	9991.4	8324.98	10063.7	10340.6	–	–	–
	0.001	PSNR	8.80749	8.07036	7.95713	8.052	8.082	7.852	8.92697	8.10322	7.98535	–	–	–
		MSE	8557.19	10140.2	10408	9126.1	9117.5	9198.6	8324.98	10063.7	10345.2	–	–	–
Salt and Pepper	0.01	PSNR	8.86333	8.05482	7.93524	–	–	–	8.85161	8.05093	7.93526	–	–	–
		MSE	8447.87	10176.5	10460.6	–	–	–	8470.71	10185.6	10460.6	–	–	–
	0.1	PSNR	8.21052	7.61238	7.51062	–	–	–	7.283	7.913	7.540	–	–	–
		MSE	9818.12	11267.9	11535	–	–	–	12155.50	10513.90	11456.60	–	–	–

### Ablation test

To demonstrate the contribution of each component in the proposed scheme, an ablation study was conducted by removing individual stages or combinations of stages from the image cryptosystem to evaluate their impact on overall performance. As illustrated in Fig. 14, simplified configurations exhibited slightly lower computational complexity; however, they resulted in noticeable degradation in security-related performance metrics. The experimental findings indicate that each module plays a significant role in enhancing randomness, reducing pixel correlation, and improving resistance against cryptographic attacks. Therefore, the results validate the effectiveness of the integrated framework and highlight the importance of incorporating all proposed components to achieve optimal encryption performance.

**Fig. 15:**
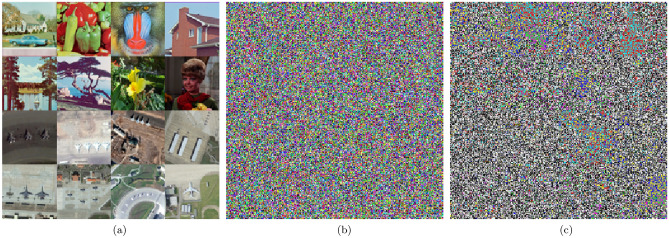
Ablation test by removing stages from the proposed scheme. (**a**) Plain Augmented. (**b**) One stage removed and (**c**) Two stages removed.

### Key sensitivity analysis

**Fig. 16:**
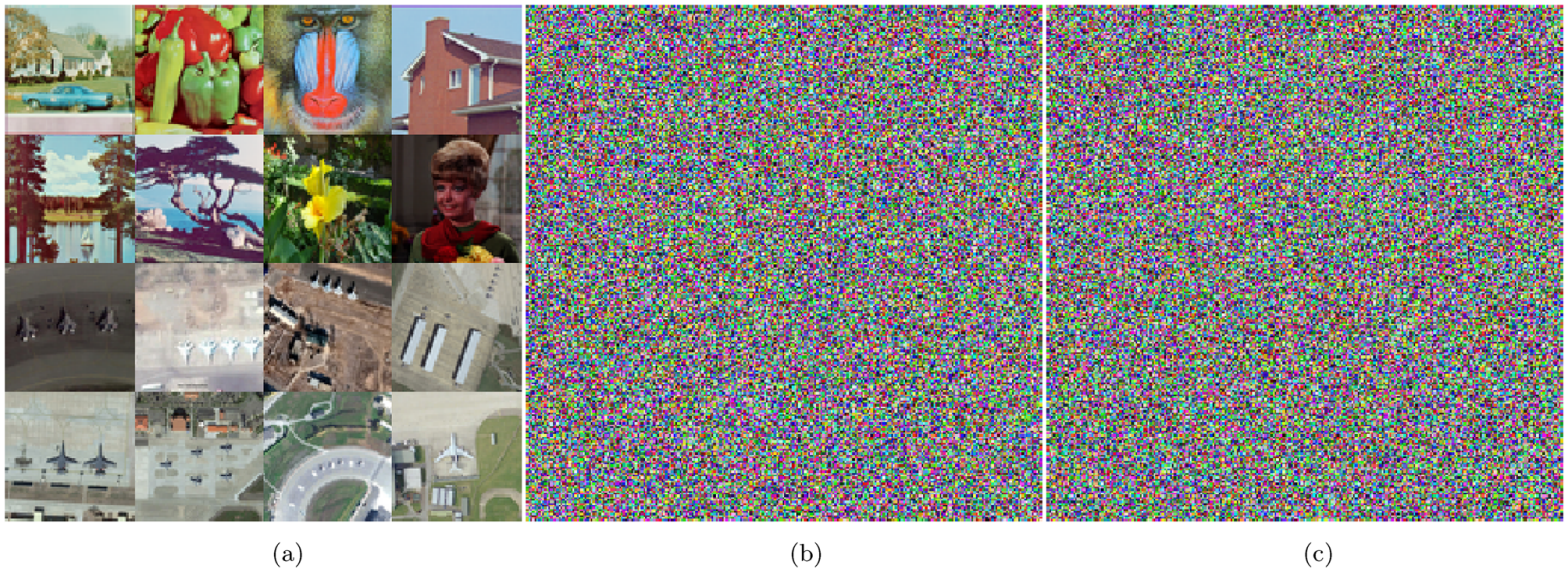
Key sensitivity analysis by decrypting images using the correct keys, a key with a difference in a single bit, or two bits. (**a**) Correct keys. (**b**) 1-bit difference and (**c**) 2-bit difference.

To demonstrate the key sensitivity of the proposed algorithm, Fig. 16 presents several decryption scenarios under slight variations of the secret key. When the correct key is used, the original image is accurately reconstructed without any distortion. However, even minimal modifications to the key, such as altering a single bit or two bits, result in completely unsuccessful decryption, producing severely distorted outputs with no resemblance to the original image. These results clearly confirm that the proposed scheme exhibits high sensitivity to small key variations, which is a desirable property for ensuring strong security against key-related attacks (Fig. 15).

### Time and memory consumption

**Fig. 17:**
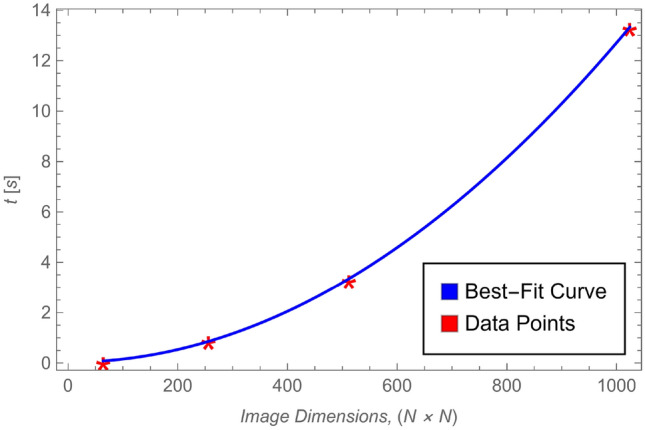
Time consumption behavior.

Figure 17 depicts the time consumption of the proposed algorithm as a function of increasing image dimensions. The results indicate that the execution time grows consistently with the image size, starting at approximately 0.06 s for a $64 \times 64$ image and reaching around 13.3 s for a $1024 \times 1024$ image. This trend suggests a predictable scaling behavior, where the computational complexity can be approximated as $O(M\times N)$ with respect to the image dimension for an $M \times N$, reflecting that the algorithm processes each pixel in a systematic manner. Consequently, the proposed approach demonstrates stable and efficient performance even when applied to high-resolution images.

**Fig. 18:**
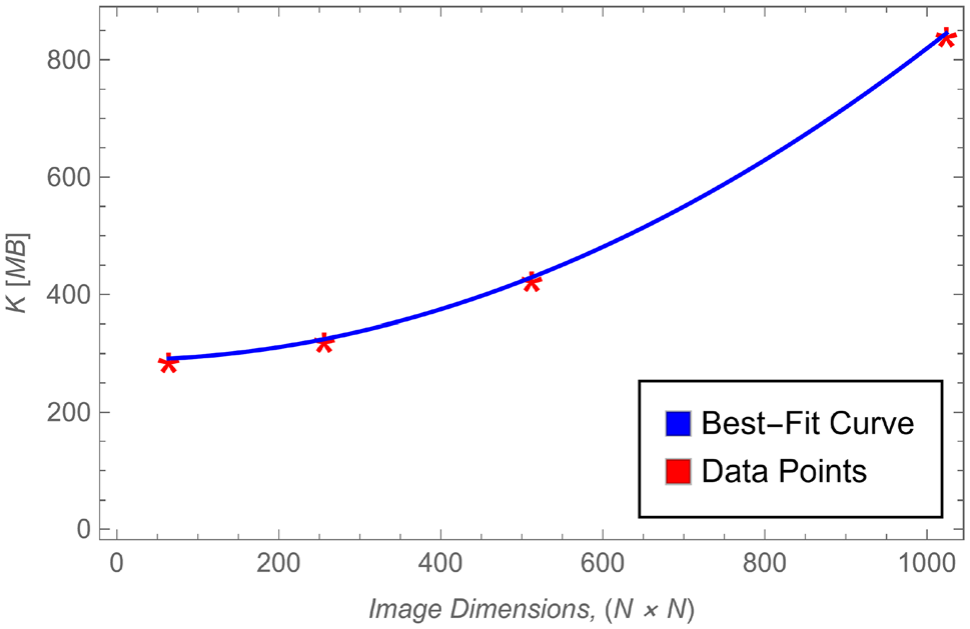
Memory consumption behavior.

Figure 18 illustrates the memory consumption of the augmented image as a function of its dimensions. The results indicate that memory usage increases approximately with image size, rising from about 300 MB for a $64 \times 64$ image to nearly 3109 MB for a $1024 \times 1024$ image. This growth behavior demonstrates that the proposed algorithm exhibits predictable and scalable memory requirements, thereby supporting its computational efficiency even when processing large-scale images.

### NIST SP 800-22 analysis

This subsection presents a comprehensive evaluation of the security properties of the proposed encryption algorithm. The robustness of the generated encrypted bitstream is rigorously assessed using the statistical test suite defined in NIST $800-22$, which is a widely recognized benchmark for evaluating the randomness of binary sequences and, consequently, the strength of encryption algorithms. For each test, a bitstream of length $10^6$, obtained by concatenating the outputs of multiple encrypted augmented images, is employed. The corresponding results are summarized in Table 11, where all tests are successfully passed, as indicated by *p*-values exceeding the threshold of 0.01. These outcomes confirm that the generated bitstream exhibits a high degree of randomness, reflecting the effectiveness of the proposed algorithm in resisting statistical attacks and reinforcing its suitability for secure cryptographic applications.

**Table 11:** Results of the NIST suite of tests.

Test	*p*-value	Conclusion
Frequency	0.909852	Success
Block Frequency	0.225792	Success
Run	0.970749	Success
Long runs of ones	0.259355	Success
Rank	0.152987	Success
Spectral FFT	0.186347	Success
Non overlapping	0.587619	Success
Overlapping	0.187166	Success
Universal	0.274961	Success
Serial	0.846168	Success
Serial	0.911109	Success
Approx. entropy	0.413316	Success
Cum. sums forward	0.852370	Success
Cum. sums reverse	0.935301	Success
Random Excursions (RE) 1	0.537129	Success
RE 2	0.036081	Success
RE 3	0.790827	Success
RE 4	0.293669	Success
RE 5	0.309498	Success
RE 6	0.493400	Success
RE 7	0.074661	Success
RE 8	0.537129	Success
Random Excursions Variant (REV) 1	0.936512	Success
REV 2	0.696482	Success
REV 3	0.724682	Success
REV 4	0.761378	Success
REV 5	0.804024	Success
REV 6	0.797534	Success
REV 7	0.433430	Success
REV 8	0.271433	Success
REV 9	0.245874	Success
REV 10	0.709734	Success
REV 11	0.685836	Success
REV 12	0.806617	Success
REV 13	0.697315	Success
REV 14	0.759202	Success
REV 15	0.328554	Success
REV 16	0.233960	Success
REV 17	0.267847	Success
REV 18	0.555565	Success

## Conclusions and future work

This work presented a multi-stage, chaos-chess hybrid cipher tailored to the security needs of high-resolution satellite imagery. Fractional-order hyperchaotic systems were employed to generate plaintext-sensitive keys and dynamic S-boxes, while Knight’s Tour-guided traversal on 8×8 blocks was used to drive bitwise diffusion. Across a diverse set of satellite and natural images, the scheme achieved near-ideal entropy, strong differential resistance (high NPCR and appropriate UACI), near-zero adjacent-pixel correlations, and a large key space, indicating robustness against statistical, brute-force, and differential attacks. Visual, histogram, and DFT analyses confirmed noise-like ciphertexts and faithful, lossless recovery. Robustness under common channel impairments and content loss (occlusion, salt-and-pepper, Gaussian noise) was also evidenced, supporting the applicability of the method to real-world remote sensing pipelines. Overall, a scalable and modular cryptosystem has been demonstrated that integrates chaos-based confusion/diffusion, algebraic mixing on 2×2 pixel matrices, and chess-inspired traversal to enhance security without sacrificing practical deployability.

Future efforts will be directed along six axes. (1) Hardware acceleration and deployment: FPGA/ASIC implementations and SIMD/GPU kernels will be investigated to quantify energy-latency trade-offs, throughput at 4K-8K resolutions and video streams^43,44^, as well as radiation tolerance for space-grade platforms^31^. (2) Standards alignment and benchmarking: conformance to community reporting checklists (e.g., standardized randomness and security test suites), expanded datasets, and head-to-head comparisons under fixed budgets (time/memory) will be pursued to enable reproducibility and fair appraisal. (3) ROI-aware and task-aware integration: selective encryption tightly coupled with object detection/segmentation for bandwidth-limited downlinks will be explored, including adaptive protection levels based on target sensitivity^45^. (4) Advanced threat modeling: resilience against chosen-plaintext/chosen-ciphertext settings, adversarial examples targeting pre/post-encryption ML models, and side-channel vectors (timing, power, EM) will be systematically evaluated; countermeasures (masking, constant-time routines) will be incorporated where needed^46^. (5) Key management and protocol design: lightweight, forward-secure key evolution, multi-image/session key derivation, and secure key embedding or escrow mechanisms suitable for distributed sensing constellations will be developed^47^. (6) Algorithmic refinements: analysis of parameter sensitivity for fractional orders, rigorous proofs for S-box properties under varying seeds, alternative tours and graph-based traversals beyond Knight’s Tour, and hybridization with post-quantum primitives for future-proofing will be conducted^48–50^. Together, these directions aim to translate the demonstrated cryptographic strengths into a deployable, standards-compliant solution for secure, real-time satellite imaging at scale.

## Data Availability

The datasets used and/or analyzed during the current study are available from the corresponding author on reasonable request (email: wassim.alexan@ieee.org).
